# Type 2 diabetes remission: multidimensional pharmacological strategies and future perspectives

**DOI:** 10.3389/fendo.2025.1687601

**Published:** 2025-12-05

**Authors:** Yang Liu, Xiaokun Gang, Xinming Liu, Wei Jiang, Yuqi Yang, Guixia Wang

**Affiliations:** 1 Department of Endocrinology and Metabolism, The First Hospital of Jilin University, Changchun, China; 2 Department of Internal Medicine, Affiliated Hospital of Jilin Medical College, Jilin, China

**Keywords:** type 2 diabetes mellitus, disease remission, pharmacological interventions, glycemic control, combination strategies

## Abstract

The high prevalence and severe complications of type 2 diabetes mellitus (T2DM) pose significant threats to public health systems worldwide. An in-depth understanding of T2DM pathophysiology has brought focused attention to pharmacological strategies for achieving disease remission. However, limited knowledge exists regarding their efficacy, patient stratification strategies, and long-term effects. By constructing an analytical framework integrating “mechanism-drug-strategies,” we explore mechanisms of action and clinical effects of multiple medications (insulin, metformin, sodium–glucose cotransporter-2 inhibitors, and glucagon-like peptide-1 [GLP-1] receptor agonists) for T2DM remission and analyze synergistic effects of combination therapies. Short-term intensive insulin therapy significantly improves β-cell function and insulin sensitivity, resulting in sustained glycemic remission in certain patients. Novel multi-target drugs (GLP-1/gastric inhibitory polypeptide dual agonists) demonstrate significant glycemic control and weight loss advantages. Non-antihyperglycemic drugs (vitamin D, sex hormones) demonstrate diabetes remission potential. Combined therapies (insulin with oral hypoglycemic agents, multitarget oral drug combinations) improve remission rates and prolong remission duration. This study systematically synthesized evidence on the multidimensional progress in pharmacological interventions for T2DM remission to support clinical practice and promote the transition of T2DM remission from a theoretical concept to individualized clinical application. Through an in-depth analysis of drug mechanisms of action and clinical research, new perspectives and strategies for optimizing treatment plans, improving diabetes remission rates, and reducing medication burdens, potentially providing important references for global T2DM management, are described. Future research focusing on long-acting combination strategies, predictive model construction, and translational medicine validation may cause a paradigm shift, from “glycemic control” to “disease remission.”

## 1 Introduction

### 1.1 Background

**Figure 1:**
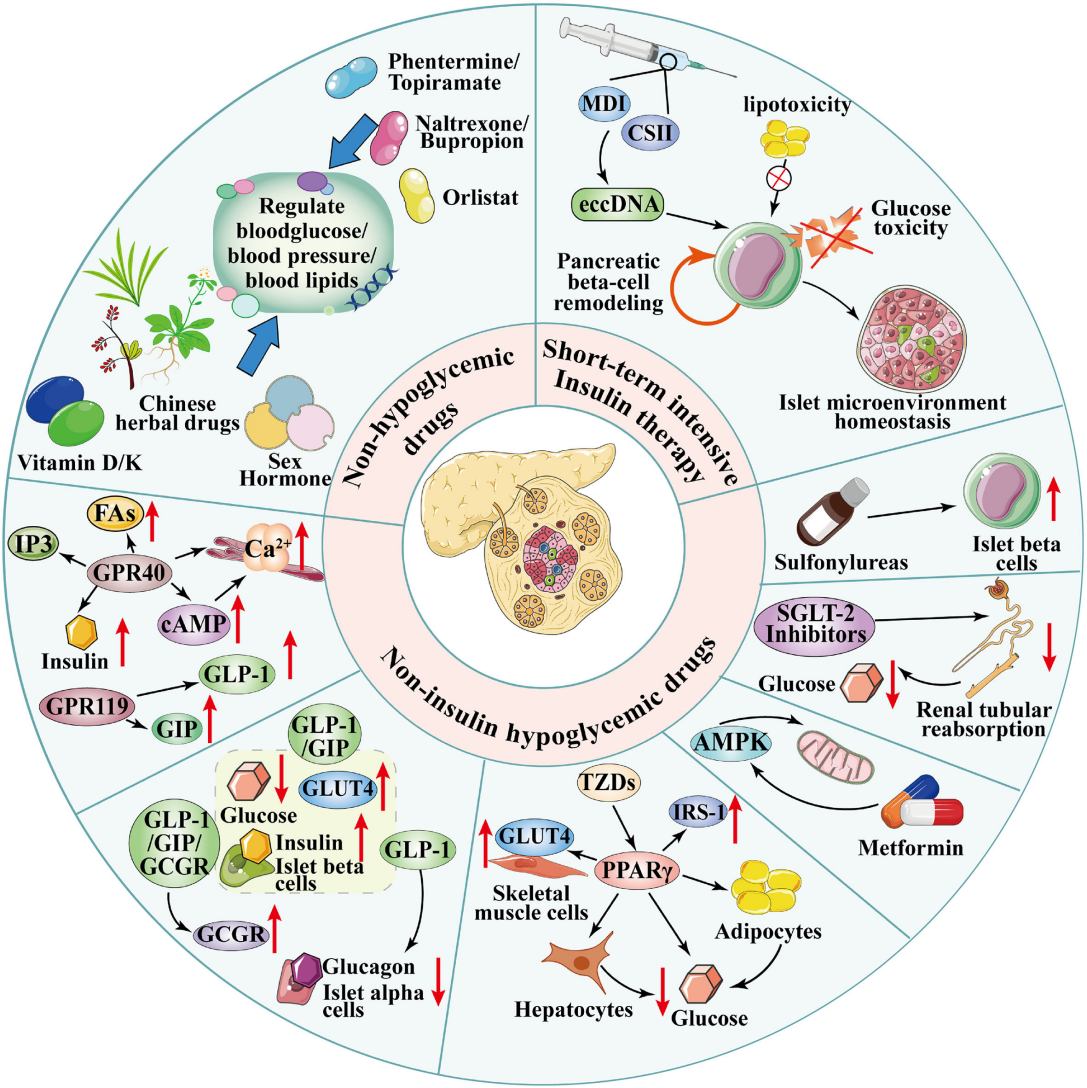
Three innovative pharmacological dimensions for type 2 diabetes mellitus (T2DM) remission. (1) Short-term intensive insulin therapy (SIIT): Delivered via continuous subcutaneous insulin infusion (CSII) or multiple daily injections (MDIs), it alleviates glucotoxicity/lipotoxicity, promotes pancreatic β-cell remodeling, and reduces plasma extrachromosomal circular DNA (eccDNA) levels to achieve sustained glycemic control. (2) Non-insulin antihyperglycemic agents: Sodium–glucose cotransporter-2 inhibitors (SGLT-2i) reduce renal glucose reabsorption; glucagon-like peptide-1 receptor agonists (GLP-1RAs) and dual/triple agonists (e.g., GLP-1/GIP, GLP-1/GIP/GCGR) regulate glucose metabolism via pancreatic (promote insulin secretion), peripheral (delay gastric emptying), and central (suppress appetite) effects; metformin activates AMP-activated protein kinase (AMPK) to improve insulin resistance; thiazolidinediones (TZDs) activate peroxisome proliferator-activated receptor gamma (PPARγ) to enhance adipocyte insulin sensitivity. (3) Non-antihyperglycemic agents: Weight-loss drugs (orlistat, phentermine-topiramate, naltrexone-bupropion) improve metabolic status via weight reduction; sex hormones (e.g., testosterone) upregulate GLUT4 expression to promote glucose uptake; vitamins (D/K) regulate insulin sensitivity through osteocalcin carboxylation or insulin receptor sensitization; traditional Chinese medicine (e.g., Jinlida granules) modulates glucose/lipid metabolism. FAs, free fatty acids; IP3, inositol trisphosphate; cAMP, cyclic adenosine monophosphate; IRS-1, insulin receptor substrate-1; GCGR, glucagon receptor; GIP, glucose-dependent insulinotropic polypeptide; GPR40/119, G protein-coupled receptors 40/119.

Type 2 diabetes mellitus (T2DM) is a critical universal public health burden. The 2019 International Diabetes Federation (IDF) report indicated that the number of 20–79 year old adults with diabetes reached 463 million (9.3% of the world’s population), with a projected increase to 700 million by 2045 (1). Over 90% of these cases are T2DM (2). Chronic complications such as renal nephropathy, retinopathy, diabetic foot, and cardiovascular complications severely threaten patient survival. In 2021, diabetes caused an estimated 6.7 million deaths, while related healthcare expenditures increased by 316% from 2007 to 2021. T2DM accounts for nearly 10% of global healthcare spending (approximately US$850 billion annually), creating an unsustainable economic burden on healthcare systems (3). In response to this escalating burden, achieving diabetes remission has become a paramount therapeutic goal (4). Studies show weight loss through bariatric surgery (5–7) or intensive lifestyle interventions (8, 9) can induce remission in overweight or obese patients with T2DM. However, long-term adherence to lifestyle modifications remains challenging for most individuals (10), and surgical risks limit patient uptake of metabolic procedures. This disparity between clinical demand and therapeutic limitations has led to a growing focus on pharmacological interventions for T2DM remission as a promising frontier. However, critical knowledge gaps persist regarding the efficacy, durability, and patient stratification strategies for pharmacologically induced remission, significantly hindering clinical translation. This review integrates the pathophysiological basis of T2DM remission and explores three innovative pharmacological dimensions: (1) repurposing traditional glucose-lowering agents by elucidating β-cell “reprogramming” mechanisms through short-term intensive insulin therapy (SIIT); (2) breakthroughs in non-insulin antidiabetic therapies, focusing on the metabolic effects of sodium–glucose cotransporter 2 inhibitors (SGLT-2i), glucagon-like peptide-1 (GLP-1) receptor agonists (RAs), and dual/triple RAs; and (3) emerging non-glycemic pharmacotherapies, assessing the potential of antihypertensives, sex hormones, herbal medicine, vitamins, and minerals in T2DM remission. Using a “mechanism–drug strategy” framework, this review aims to guide personalized treatment decisions, expedite clinical translation of T2DM remission, and improve public health outcomes by reducing polypharmacy. Regarding diabetes remission, the three innovative pharmacological dimensions mentioned above are summarized in **Figure 1**.

### 1.2 Literature selection criteria

This review explores “multidimensional pharmacological strategies for type 2 diabetes mellitus (T2DM) remission.” To maintain relevance, timeliness, and scientific integrity, core literature was selected from Chinese and English-language studies. English sources were primarily identified through PubMed and Web of Science by searching key terms like “type 2 diabetes mellitus remission” and “pharmacological intervention for T2DM remission.” Priority was assigned to original research—particularly randomized controlled trials (RCTs) and cohort studies—as well as landmark reviews. Chinese-language sources were accessed via the China National Knowledge Infrastructure (CNKI), prioritizing authoritative journal articles related to “Chinese herbal intervention in T2DM remission” and “vitamins/sex hormones and glucose metabolic regulation.”

Only studies that referenced standardized definitions for T2DM remission or provided a thorough analysis of mechanisms such as β-cell function restoration and improved insulin sensitivity were included. Studies were excluded if they lacked adequate data—such as less than three months of follow-up—or those not peer-reviewed, like conference abstracts.

Two endocrinology specialists independently reviewed the literature based on established criteria, including topic relevance, study design rigor, and clinical impact. Recent publications (2015–2025) were prioritized to capture the latest advances. Preference among clinical studies was given to those with rigorous design and robust analyses, while mechanistic studies were assessed for reproducibility and sound validation. In total, 114 core publications were selected, encompassing various interventions, including short-term intensive insulin therapy, non-insulin antihyperglycemic agents (e.g., SGLT-2 inhibitors and GLP-1 receptor agonists), and adjunctive therapies (e.g., vitamin D, sex hormones, and Chinese herbal compounds). Cumulatively, this body of literature provides a comprehensive and representative analysis of multidimensional pharmacological strategies for T2DM remission.

## 2 Insulin intensive therapy-induced diabetes remission: mechanistic insights and clinical translation

### 2.1 Definition and criteria for the assessment of diabetes remission

Diabetes remission is defined as the sustained glycemic control (glycated hemoglobin [HbA1c] < 6.5%) achieved over a clinically significant period after reducing or discontinuing glucose-lowering medications. The American Diabetes Association (ADA) defines remission of T2DM as follows: (1) maintaining an HbA1c of < 6.5% (< 48 mmol/mol) for at least 3 consecutive months without pharmacologic therapy; and (2) when HbA1c measurements are unreliable, alternative criteria include fasting plasma glucose (FPG) < 7.0 mmol/L or 126 mg/dL, or estimated HbA1c from continuous glucose monitoring (CGM) < 6.5% (11).

### 2.2 Core mechanisms of SIIT

SIIT is a validated strategy for T2DM reversal (12). Delivered via continuous subcutaneous insulin infusion (CSII) or multiple daily injections (MDIs), SIIT achieves rapid glycemic normalization through two synergistic mechanisms, metabolic detoxification and β-cell functional remodeling. Chronic hyperglycemia-induced glucotoxicity and lipotoxicity drive β-cell dedifferentiation and dysfunction (13). SIIT interrupts this cycle, reducing glucose/lipid toxicity to restore insulin sensitivity and promote β-cell redifferentiation (14, 15). Newly diagnosed patients undergoing SIIT show elevated plasma extrachromosomal circular DNA (eccDNA) at 1-year follow-up, whereas patients achieving remission maintain significantly lower eccDNA loads compared to non-responders (*P* < 0.05), suggesting a potential association between eccDNA dynamics and sustained glycemic control (16). These findings provide the basis for the potential future application of eccDNA in monitoring the progression of T2DM and predicting the response of newly diagnosed patients with diabetes to SIIT treatment.

### 2.3 Clinical efficacy and predictive factors

A recent multicenter RCT provides strong support for early intensive insulin therapy to help newly diagnosed patients with T2DM achieve remission. The study comprised 412 patients with newly diagnosed T2DM and severe hyperglycemia (mean HbA1c: 11.0%), all of whom received SIIT for two to three weeks. In the control group, which only adopted lifestyle interventions without further medication, nearly half (48%) maintained their HbA1c below 6.5% after 48 weeks—a result classified as successful diabetes remission by current international standards. Early SIIT was found to significantly improve β-cell function and insulin sensitivity, laying the foundation for long-term glycemic control without medication for some patients (17).

Similarly, a study with 48 newly diagnosed patients with T2DM, all of whom received a 2-week course of SIIT, reported the following outcomes at the 1-year follow-up after treatment cessation: a notable decline in the Homeostatic Model Assessment of Insulin Resistance (HOMA-IR), a significant increase in the Homeostatic Model Assessment for β-cell function (HOMA-β), and improvement in the acute insulin response (AIRins). The near-normal recovery of insulin sensitivity induced by SIIT may be a key factor contributing to long-term glycemic control and potential remission (18) (small-scale RCT [*n* = 48, 1-year follow-up]). The influence of baseline body mass index (BMI) on short-term and long-term glycemic remission is complex. In a study of 124 drug-naive patients with T2DM receiving 2 weeks of CSII, first-phase insulin secretion recovered, and glucose infusion rate (GIR) significantly improved (*P* < 0.0001). At the 3-month follow-up, patients with a baseline BMI of ≥ 25 kg/m² had higher short-term remission rates and greater improvements in GIR and AIRins than lean patients. However, long-term follow-up at 6, 12, and 24 months showed no significant differences in glycemic remission rates compared with the two other groups. This indicates that baseline BMI affects short-term remission, whereas long-term remission depends more on insulin sensitivity and β-cell function (19) (medium-scale RCT [*n* = 124, 2-year follow-up]).

Further mechanistic insights suggest that SIIT can also lower glucagon levels and improve alpha-cell function (20) (medium-scale RCT [*n* = 108, 1-year follow-up]), further supporting islet function restoration. This recent high-quality evidence confirms that early SIIT is a promising strategy for achieving true drug-free remission. Indeed, a growing body of literature consistently points to a restoration of metabolic balance—primarily through reduced glucose toxicity and recovery of β-cell function—as the primary mechanism underpinning these improvements.

### 2.4 Existing challenges and future directions

Despite the widely recognized efficacy of SIIT in T2DM remission, numerous challenges impede its clinical application. Future optimization is required in the following areas.

#### 2.4.1 Optimization of treatment protocols

The duration of SIIT in patients with recently diagnosed or short-duration T2DM ranges from 2 weeks to 3 months. Most protocols maintain glycemic control for 2 weeks after reaching the target glucose levels, with a total course of 2–3 weeks. However, some patients may require extended treatment, lasting 4–12 weeks, to achieve adequate recovery. The optimal duration and whether extended courses improve diabetes remission rates remain inconclusive. Future efforts should focus on establishing individualized protocols based on dynamic monitoring of β-cell function. Additionally, further research is needed to compare various intensive treatment options, including insulin monotherapy, insulin combined with other antihyperglycemic agents, basal insulin therapy, basal insulin with GLP-1 receptor agonists (RAs), or sequential treatment. The potential benefits of pulsed SIIT (e.g., intensification–remission–cessation, relapse–re-intensification) for reversing diabetes also require more clinical evidence.

#### 2.4.2 Combination therapy strategies

In newly diagnosed patients with T2DM, SIIT typically achieves rapid glycemic control without the need for additional antihyperglycemic agents. However, some patients may benefit from combination therapy with metformin, acarbose, thiazolidinediones (TZDs), SGLT-2i, and GLP-1RAs. Insulin secretagogues are not recommended during SIIT. Current evidence is mostly derived from small-scale studies; therefore, large-scale clinical trials are needed to rigorously assess the safety and efficacy of combination therapy strategies (**Table 1**).

**Table 1:** Efficacy of diabetes remission with major antihyperglycemic drugs and combination therapy.

Drug/regimen	Mechanism of action	Remission outcomes (key data)	References
Short-term intensive insulin therapy	Relieving glucolipotoxicity and restoring β-cell function.	48% of patients maintained glycated hemoglobin (HbA1c) < 6.5% at 48 weeks.	(17)
Thiazolidinediones (TZDs)	PPARγ activation, improving insulin sensitivity in adipose tissue.	In the pioglitazone group, 57.9% of patients had HbA1c ≤ 6.5% after discontinuation of the drug; the duration of maintaining HbA1c at < 6.2% was longer than the comparison group.	(21)
Novel PPAR pan- agonist	Multitarget regulation of glucose and lipid metabolism (e.g., Sigenatide).	In the Sigenatide group, HOMA-IR and HOMA-β were significantly improved, with significant potential for remission.	(22, 23)
Comprehensive Strategy (Lifestyle, Glargine, Lixisenatide, & Metformin)	Synergistically improve β-cell function and sensitivity.	43% reduction in diabetes relapse risk. Remission rates approximately doubled at 24 weeks (RR: 1.92) and 36 weeks (RR: 1.83) after drug withdrawal.	(24)
Dual/triple receptor agonist	Multitarget regulation (GIP/GLP-1/GCGR).	Tirzepatide: In the 15 mg group, HbA1c decreased by 2.07%, and body weight decreased by 9.5 kg; the remission rate of prediabetes was 95.3% vs. 61.9% (placebo).	(25, 26)

CSII, continuous subcutaneous insulin infusion; AMPK, AMP-activated protein kinase; PPARγ, peroxisome proliferator-activated receptor gamma; HbA1c, glycated hemoglobin; HOMA-IR, Homeostatic Model Assessment for Insulin Resistance; HOMA-β, HOMA for Beta-cell function; GIP, glucose-dependent insulinotropic polypeptide; SIIT, short-term intensive insulin therapy; GLP-1, glucagon-like peptide-1; GCGR, glucagon receptor; cAMP, cyclic adenosine monophosphate; PKA, protein kinase A.

#### 2.4.3 Development of predictive models

Currently, no predictive models accurately identify patients most likely to benefit from SIIT. Future research should integrate multi-omics data (e.g., metabolomics, epigenetic markers) and apply machine learning algorithms to create individualized predictive models. These models can be used to evaluate treatment responses, optimize protocols, increase diabetes remission rates, and underpin personalized therapy.

#### 2.4.4 Considerations for clinical translation

Although SIIT has proven effective in achieving diabetes remission, several practical barriers hinder its real-world implementation. Cost-effectiveness remains a concern, particularly in resource-limited settings, due to the costs of continuous glucose monitoring and insulin pump therapy. Access to specialized care teams and diabetes education programs also varies globally, impacting how widely SIIT can be implemented. Additionally, factors such as socioeconomic status, health literacy, and cultural background can influence patient adherence to frequent injections or pump usage. SIIT also carries potential risks such as hypoglycemia and weight gain, necessitating careful patient selection and monitoring. Future research should focus on simplifying treatment protocols, training primary care providers, and integrating digital health tools to support feasibility and scalability.

## 3 Remission of T2DM with non-insulin antihyperglycemic therapies

Novel non-insulin antihyperglycemic drugs with dual glycemic control and weight-reducing effects are increasingly recognized for their potential to promote T2DM remission (27, 28). Among them, metformin, SGLT-2 inhibitors (SGLT-2i), thiazolidinediones (TZDs), GLP-1 receptor agonists (GLP-1RAs), and dipeptidyl peptidase 4 (DPP-4) inhibitors represent the most promising candidates due to their multiple beneficial effects beyond glucose lowering (**Table 1**).

### 3.1 Metformin: more than lowering blood sugar—a multidimensional strategy for achieving diabetes remission

There is currently a lack of large-scale randomized controlled trials that strictly adhere to the modern definition of drug-free remission. Metformin is administered as a first-line therapy according to the ADA guidelines for T2DM, favored for its potent glycemic control, good tolerability, high safety, and low cost (29). Recent research has shown that it can also induce weight loss, lower cancer incidence and mortality, and potentially extend lifespan (30). Moreover, in combination with other antihyperglycemic medications, metformin significantly increases the remission rate in diabetes. Metformin improves insulin resistance by activating AMP-activated protein kinase (AMPK), which regulates mitochondrial fission and function (31), thereby alleviating hyperglycemia. Its pleiotropic effects (32) provide a unique advantage for diabetes remission.

#### 3.1.1 Core principles behind approaches for sustained remission

Maintaining long-term diabetes remission remains a significant challenge. Randomized trials support the role of metformin as a cornerstone therapy for sustaining remission. For example, a prospective randomized trial found that metformin (1000 mg/day) reduced the risk of hyperglycemia recurrence by approximately 70% and extended the median remission duration from 10 to 16 months in 48 African American patients with obesity who attained near-normoglycemic remission after SIIT. Improved β-cell function was confirmed as the mechanism underlying these results (33) (small-scale RCT [*n* = 48, 4-year follow-up]). This study’s design—administering metformin after initial remission—supports the approach of maintaining a drug-free state, thereby highlighting its unique value within the remission paradigm.

#### 3.1.2 Integrated elements of comprehensive remission approaches

Recently, comprehensive treatment strategies have shown considerable promise in promoting diabetes remission. The key RCT (24), offered robust evidence supporting this approach. Participants underwent a 12-week intensive metabolic intervention combining lifestyle changes, basal insulin or GLP-1 receptor agonists, and metformin. The primary outcome was diabetes remission—defined as an HbA1c below 6.5% after discontinuing all antihyperglycemic medications. Those receiving the comprehensive intervention experienced remission rates at 24 and 36 weeks that were approximately double those observed in the standard care group. Hence, a structured, short-term regimen combining metformin as a foundational element with intensive agents like insulin/GLP-1RAs and lifestyle modifications, acts synergistically to markedly improve remission rates. Metformin notably enhances insulin sensitivity, helps reverse glucotoxicity, and aids in recovering β-cell function, making it a crucial component of this therapeutic strategy.

### 3.2 SGLT-2i: an integral component in combination therapies aimed at achieving remission

SGLT-2i, selectively expressed in kidneys, act on the proximal tubules to decrease renal glucose reabsorption. This promotes urinary glucose excretion, reducing blood glucose levels, independent of insulin function (34). The process also alters systemic energy metabolism; because urinary glucose loss depletes available glucose, the body shifts to fat as the main energy source, reducing carbohydrate reliance. This triggers increased fat breakdown in the liver, with fatty acids converted to ketone bodies for energy, such as β-hydroxybutyrate. Such metabolic restructuring lowers blood glucose levels, as well as benefits cardiovascular and renal health (35). The potential for diabetes remission is related to this systemic metabolic change. Currently, no clinical studies have confirmed that SGLT-2i monotherapy can induce T2DM remission. However, the Remission Evaluation of Metabolic Interventions in Type 2 Diabetes with Dapagliflozin study reported that short-term intensive metabolic intervention increases remission rates. The relative risks for diabetes remission at 36, 48, and 64 weeks were 2.4, 2.1, and 1.8, respectively; having reduced the relapse risk by 43% (27) (medium-scale RCT [*n* = 154, maximum follow-up 1.2 years]). Although the glucose-lowering efficacy of SGLT-2i alone is limited, its combination with other drugs may enhance remission effects. Further research is required to optimize combination therapy regimens.

### 3.3 TZDs: adipose tissue restructuring and diabetes remission

TZDs are highly selective for peroxisome proliferator-activated receptor gamma (PPARγ). The PPARγ activation enhances insulin sensitivity in adipocytes, skeletal muscle, and macrophages, and inhibits hepatic gluconeogenesis, thus lowering blood glucose levels. TZDs have advanced significantly as a T2DM treatment (36).

#### 3.3.1 Remission potential and mechanistic basis of TZDs

Early clinical investigations into TZD have explored the possibility of achieving diabetes remission through treatment cessation. For example, a study involving Japanese patients with early-stage T2DM compared the long-term effects of pioglitazone and sulfonylureas. Although sulfonylureas delivered superior glucose-lowering efficacy during the active phase of treatment, patients in the pioglitazone group maintained ideal blood glucose levels (e.g., < 6.2%) for a significantly longer period during the post-discontinuation observation phase. This outcome suggests that pioglitazone, by improving insulin resistance, may foster a metabolic environment conducive to sustained remission following drug withdrawal (21) (medium-scale RCT [*n* = 278, 18-month follow-up]).

While large-scale RCTs that strictly adhere to the modern consensus definition of drug-free remission are lacking, current evidence highlights the distinct potential of TZDs to support prolonged glycemic recovery. TZDs exert powerful dual effects: significantly enhance insulin sensitivity and preserve β-cell function. These mechanisms are recognized as pivotal for achieving durable glycemic control. Hence, pioglitazone is considered a highly promising candidate for inclusion in combination strategies aimed at inducing diabetes remission.

#### 3.3.2 Breakthrough with novel PPAR pan-agonists

Chiglitazar sodium, a novel non-TZD PPAR pan-agonist, represents a new approach to diabetes remission strategies due to its distinctive mechanism of action. Clinical trials, including a large-scale RCT (*n* = 535), have demonstrated that chiglitazar sodium effectively reduces HbA1c, improves fasting plasma insulin, and enhances measures of β-cell function (HOMA-β) and insulin resistance (HOMA-IR) (22). Comparative studies further indicate that chiglitazar sodium’s efficacy is non-inferior or even superior to conventional agents like sitagliptin (23).

The unique action of chiglitazar sodium involves balanced activation of PPARα, γ, and δ subtypes, conferring both glucose-lowering and lipid-modifying effects. This multi-targeted approach comprehensively ameliorates metabolic disorders, aligning closely with the multi-factorial pathophysiological corrections required for diabetes remission. Although direct evidence supporting its ability to maintain normoglycemia after drug withdrawal according to current consensus definitions of remission is lacking, chiglitazar sodium’s pronounced effects on improving insulin sensitivity and preserving β-cell function position it as a leading candidate for future research targeting diabetes remission.

### 3.4 GLP-1RAs: multidimensional regulation and diabetes remission

Distal intestinal L-cells secrete GLP-1 and proximal K-cells release glucose-dependent insulinotropic peptide (GIP), both of which regulate pancreatic β-cell activity after nutrient intake (37). GLP-1RAs lower blood glucose by (1) promoting glucose-dependent insulin secretion and suppressing glucagon, thus reducing hypoglycemia risk; (2) slowing gastric emptying to mitigate post-prandial glucose spikes; and (3) suppressing appetite via central nervous system effects, leading to weight loss (38, 39). These diverse mechanisms make GLP-1RAs promising for diabetes remission.

#### 3.4.1 Clinical applications and approvals

The U.S. Food and Drug Administration (FDA) has approved several GLP-1RAs for T2DM treatment, including exenatide, liraglutide, dulaglutide, albiglutide, lixisenatide, semaglutide, and tirzepatide (40). Their use has expanded from glycemic control to weight management and other metabolic benefits, and recently to metabolic liver disease, peripheral arterial disease, and neurodegenerative disorders (41). As our understanding of the incretin pathway deepens, novel GLP-1-based molecules continue to emerge, including dual and triple agonists. These include molecules targeting multiple receptors, such as GCGR-GLP1R dual agonists (e.g., survodutide, pemvidutide); GCGR-glucose-dependent insulinotropic polypeptide receptor (GIPR)-GLP1R triple agonists (e.g., retatrutide); and GIPR-GLP1R dual agonists (e.g., tirzepatide). Those with optimized delivery, including small-molecule oral GLP-1RAs (e.g., oral semaglutide) and small-molecule oral GIPR-GLP1 dual agonists (e.g., orforglipron). Others are combination therapies, including pairings such as GLP-1RAs with long-acting amylin receptor (AMLNR) agonists (e.g., cagrilintide) (42, 43). These advancements broaden the patient population for GLP-1RA therapy and highlight their potential to achieve T2DM remission through mechanisms such as improved β-cell function, reduced insulin resistance, and weight loss, potentially surpassing current treatment paradigms.

#### 3.4.2 GLP-1RA monotherapy: minimal evidence for sustained remission

As of June 2025, a review of RCTs indexed in PubMed reveals no consensus-level studies investigating GLP-1RAs as monotherapy for diabetes remission, when using the primary endpoint of “maintaining HbA_1_c < 6.5% without pharmacotherapy for at least 3 months.”

To address this evidence gap, our research team has initiated a randomized, controlled, open-label clinical trial aimed at systematically evaluating the efficacy of the GLP-1RA exenatide (administered via injection) versus premixed insulin (NovoRapid 30) in inducing clinical remission among treatment-naive patients with overweight or obese T2DM. The results from this study are expected to provide critical insights into the potential of GLP-1RA to induce diabetes remission.

#### 3.4.3 Combination therapies: synergistic effects and limitations

Key evidence for the synergistic benefits of combination therapies has been provided by an RCT designed to induce remission. A 12-week short-term intensive intervention—combining lifestyle management, basal insulin (glargine), a GLP-1RA (lixisenatide), and metformin—was compared to standard care. This comprehensive intervention significantly reduced the risk of diabetes relapse by 43% compared with the standard care group. Furthermore, at 24 and 36 weeks after drug withdrawal, the intensive therapy approximately doubled diabetes remission rates as defined by consensus criteria, yielding relative risks of 1.92 and 1.83, respectively (24).

Hence, combining GLP-1RAs with insulin has emerged as a promising approach for achieving diabetes remission. However, the efficacy of this strategy depends heavily on the specific GLP-1RA formulation and the therapy duration. Notably, combinations involving short-acting GLP-1RAs and basal insulin have demonstrated significant limitations.

A small-scale RCT (*n* = 109, 20-week follow-up) conducted by Retnakaran et al. (44) investigated whether adding exenatide to basal insulin therapy could improve β-cell function and promote diabetes remission. Although the addition of exenatide was beneficial for glycemic control during treatment, it did not significantly enhance β-cell function or the likelihood of long-term diabetes remission. Similar patterns were observed in earlier studies where sequential administration of a GLP-1RA followed SIIT.

A medium-scale study (*n* = 129, 2-year follow-up) by Shi et al. (45) applied a remission definition of “HbA1c < 7.0% without glucose-lowering medications.” In patients with new-onset diabetes, those receiving 12 weeks of exenatide following a 3-week intensive insulin pump regimen had significantly higher cumulative remission rates at one and two years (68.2% and 53.0%, respectively) compared to those in the insulin-only group (36.5% and 31.8%, respectively). However, this therapeutic advantage was confined to the treatment period; upon discontinuation of exenatide, the remission maintenance curves between the two groups converged.

In summary, while combining a GLP-1RA with insulin represents a valid pathway to achieving diabetes remission, its efficacy depends on selecting an appropriate GLP-1RA formulation and treatment duration. Future research should aim to optimize key variables in this combination strategy—including agent selection, treatment duration, and personalized approaches.

#### 3.4.4 Controversies and insights on post-treatment effects

The post-treatment effects of GLP-1RAs remain controversial. A study (46) (small-scale RCT [*n* = 69, 68-week follow-up]) compared insulin glargine and exenatide in patients with T2DM. Exenatide significantly enhanced β-cell function, specifically in the first and second stages of insulin excretion. The exenatide group lost 3.6 kg, whereas the insulin glargine group gained 1.0 kg, resulting in a 4.6 kg difference between the groups (*P* < 0.0001). After treatment, reductions in HbA1c levels were similar between groups (*P* = 0.55). However, 4 weeks after discontinuation, β-cell function and glycemic control returned to baseline in both groups, suggesting that the drug benefits were not sustained. A 3-year study (47) [A small clinical study (*n* = 69, 160-week follow-up)] evaluated patients treated with exenatide and insulin glargine and observed post-discontinuation effects. After 3 years, both drugs showed similar glycemic control, but exenatide significantly reduced body weight (*P* < 0.001). The disposition index (DI), reflecting insulin sensitivity and functionality of pancreatic β-cells balance, increased in the exenatide group post-discontinuation (1.43 ± 0.78) compared to a decrease in the insulin glargine group (-0.99 ± 0.65, *P* = 0.028), and remained elevated 4 weeks after stopping exenatide, indicating β-cell benefits. This study suggests that exenatide has lasting positive effects on pancreatic β-cell function and weight in T2DM, supporting the long-term benefits of GLP-1RAs on β-cells. Discrepancies between studies may be due to population heterogeneity (e.g., disease duration, BMI, residual β-cell function), implying baseline metabolic traits affect drug durability. Further research is required on the mechanisms underlying post-treatment effects and their impact on long-term glycemic control.

#### 3.4.5 Breakthroughs with novel multi-target agonists

The advent of dual- and triple-receptor agonists marks a new era for T2DM remission. Multi-target drugs like tirzepatide, a dual-targeting agonist that binds to and activates the GLP-1R and GIPR, enhance insulinotropic responses, thereby improving glycemic control. By synergistically activating downstream pathways (e.g., cAMP–PKA/Epac2), they improve β-cell function, suppress appetite, and regulate lipid metabolism, thereby expanding T2DM treatment options (48).

##### 3.4.5.1 Efficacy of tirzepatide

A study comparing tirzepatide and semaglutide in patients with T2DM showed that greater effectiveness in reducing HbA1c and achieving weight loss was observed with tirzepatide treatment (both *P* < 0.001) (49). The SURPASS-1 study (large-scale RCT [*n* = 478, 40-week follow-up]) found that tirzepatide monotherapy significantly decreased HbA1c levels: the patient groups receiving 5, 10, and 15 mg doses showed decreases of 1.87%, 1.89%, and 2.07%, respectively (25 HbA1c targets of < 6.5% were achieved by 81–86%, and < 7.0% by 87–92%, with 31–52% reaching < 5.7%. Weight loss was dose-dependent (7.0–9.5 kg), and all tirzepatide groups outperformed the placebo in FPG, HbA1c levels, HbA1c targets, and weight reduction. In a large-scale RCT for obesity (*n* = 2,539, 72-week follow-up), 95.3% of participants that received tirzepatide achieved normoglycemia (26), compared to 61.9% in the placebo group. These data suggest that tirzepatide has notable therapeutic effects on obesity, as well as aids in achieving prediabetes remission. Tirzepatide improved glycemic control and weight loss without increasing hypoglycemia risk, with a safety profile analogous to that of GLP-1RAs, suggesting its potential as a monotherapy for T2DM remission.

##### 3.4.5.2 Potential of triple receptor agonists

In 2015, a preclinical study was designed to validate a novel single-molecule triple receptor agonist targeting GIPR, GLP-1R, and GCGR in diet-induced obese (DIO) mice (50). This triple agonist induced greater weight loss in DIO mice than the dual GIPR/GLP-1RA agonist, without further reduction in food intake. Retatrutide (LY3437943), the first GIP/GLP-1/GCGR triple receptor agonist to enter clinical development, demonstrated significant weight reduction—up to 24.2% in the highest dose group—in a phase 2 clinical trial. This degree of weight loss is rare among previous anti-obesity drug trials and is comparable to outcomes achieved through bariatric surgery (e.g., gastric bypass). Notably, participants experienced continued weight loss throughout the study period without reaching a plateau (51) (medium-scale RCT [*n* = 338, 48-week follow-up]). In phase 2 trials, retatrutide’s weight loss effect (-24.2%, *P* < 0.001) was comparable to that of metabolic surgery. As bariatric surgery effectively induces T2DM remission, further research is required to determine whether retatrutide can promote diabetes remission at similar rates and to define its role in diabetes remission.

#### 3.4.6 Innovations in drug delivery

The emergence of oral non-peptide GLP-1RAs, including orforglipron, represents a crucial breakthrough in therapeutic strategies. A trial showed that orforglipron induced significant weight loss in obese adults, with continuous reduction up to 36 weeks (52) (medium-scale RCT [*n* = 272, 36-week follow-up]). Another study found that in patients with T2DM, orforglipron at 12 mg or higher doses reduced HbA1c and body weight more effectively than placebo and dulaglutide (53) (medium-scale RCT [*n* = 383, 26-week follow-up]). Its safety profile mirrored that of phase-matched GLP-1RAs. Injectable GLP-1RAs and oral semaglutide could be replaced by orforglipron, potentially enhancing patient adherence and providing new options for T2DM remission.

#### 3.4.7 Summary and future perspectives

**Figure 2:**
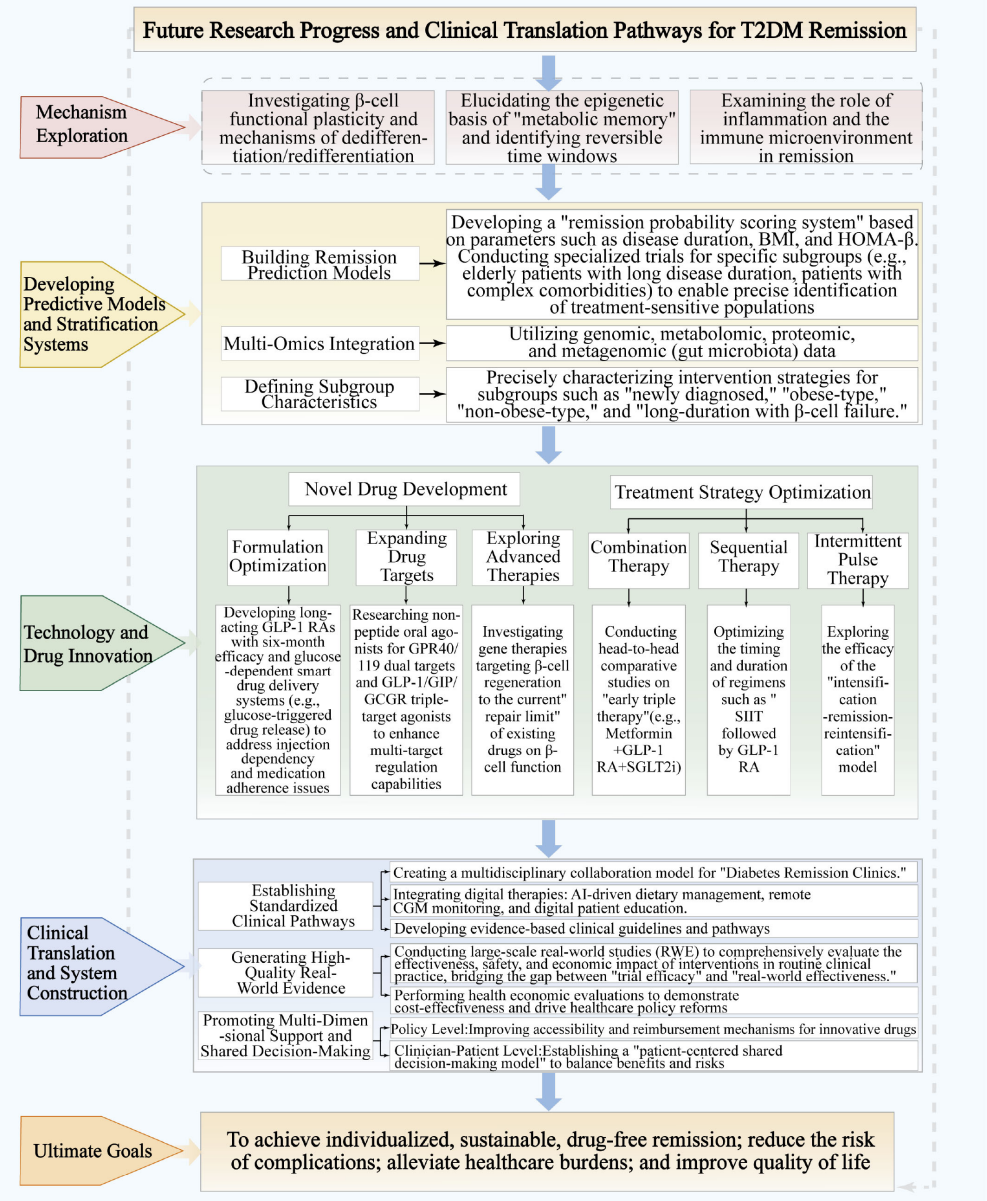
Future research progress and clinical translation pathways for T2DM remission.

GLP-1RAs are key for T2DM remission via multi-target regulation, including improving β-cell function, controlling weight, and enhancing insulin sensitivity. However, continuous treatment is required to sustain these effects. Future research should utilize omics technologies to identify optimal beneficiary populations, elucidate the molecular mechanisms of post-treatment effect heterogeneity, and develop smart drug delivery systems for personalized therapy (**Figure 2**). With the emergence of multi-receptor agonists and oral formulations, pharmacological treatments may soon match or exceed the remission rates of metabolic surgery, potentially transforming the treatment paradigm for T2DM.

### 3.5 Synergistic effects and mechanistic exploration of combination therapies

#### 3.5.1 Insulin and OHA: value of early intensive intervention

Early initiation of combination therapy involving insulin and OHAs has demonstrated enhanced remission rates in T2DM. Clinical evidence from a small-scale RCT (*n* = 129, 52-week follow-up) shows that 37.9% of patients receiving combined treatment can achieve glycemic remission after one year, compared to 20.9% in those treated with OHAs alone, highlighting the superiority of the combination approach (54).

Further supporting the benefits of intensive metabolic intervention, a representative study found that short-term lifestyle and pharmacological therapy (including metformin, acarbose, and insulin glargine) led to normoglycemia rates of 50.0% and 70.4% at 8 and 16 weeks, respectively, while the control group achieved only 3.6%. Three months after the intervention, remission rates were 21.4% in the 8-week group and 40.7% in the 16-week group (55) (small clinical study [*n* = 83, 52-week follow-up]). These findings underscore the potential of short-term, intensive combination therapy to induce diabetes remission.

Recent research, such as the REMIT study series, has further refined this approach. The REMIT-DAPA study indicated that the short-term intensive intervention group (including lifestyle changes and insulin glargine, metformin, and dapagliflozin) had a 24.7% diabetes remission rate at 24 weeks (vs. 16.9% in the control group). Although not statistically significant at 24 weeks, the intervention group had relative remission risks of 2.4, 2.1, and 1.8, in contrast to the control group at 36, 48, and 64 weeks, respectively (27) (medium-scale RCT [*n* = 154, 64-week follow-up]).

Similarly, the REMIT-SITA trial showed that at 36 weeks, in the intensive intervention group (which included insulin glargine, sitagliptin/metformin, and lifestyle therapy), 26% of participants who had HbA1c level of < 7.3% at 12 weeks remained in remission post-discontinuation, compared to 10% among the control participants (56) (small-scale RCT [*n* = 102, 52-week follow-up]). This research yields preliminary findings for a new T2DM treatment strategy, indicating that intensive therapy may induce remission and guide future research.

Exploration of synergistic effects between CSII and pharmacologic agents with distinct mechanisms—such as rosiglitazone, metformin, or alpha-lipoic acid—has important implications for optimizing intensive therapy regimens. One study reported near-normal glycemia rates at 3 months as follows: 72.5% (CSII), 87.5% (CSII + rosiglitazone), 90% (CSII + metformin), and 75% (CSII + α-lipoic acid). The CSII + metformin group achieved normoglycemia more quickly, required less insulin, and exhibited greater improvements in cholesterol levels, insulin response, and β-cell function. The CSII + rosiglitazone group was more effective at reducing IMCL. CSII + α-lipoic acid had similar outcomes to CSII alone. Findings indicate that combining CSII with metformin or rosiglitazone is more beneficial than CSII monotherapy, whereas α-lipoic acid adds no advantage (57) (medium-scale RCT [*n* = 160, 12-week follow-up]).

Collectively, these studies suggest that combining CSII with insulin-sensitizing agents may alleviate insulin resistance and glucolipotoxicity, creating a more favorable metabolic milieu for β-cell function restoration. Although direct assessment of diabetes remission as an endpoint has not been performed, the mechanistic synergies observed offer a robust theoretical foundation and clear direction for future trials specifically designed to achieve remission using CSII-based combination regimens.

#### 3.5.2 Novel drug combinations: mechanistic synergy and clinical prospects

##### 3.5.2.1 Combination therapy (metformin+GLP-1+TZD)

A pathophysiology-guided strategy targeting fundamental defects in T2DM—such as the combination of metformin, a GLP-1RA, and a TZD—represents a promising approach for inducing diabetes remission. Evidence from a medium-scale (*n* = 221; 2-year follow-up) landmark trial demonstrated that patients with newly diagnosed T2DM who received early intensive triple therapy (metformin/pioglitazone/exenatide) achieved superior and sustained glycemic control compared to those managed with conventional stepwise therapy (58). Notably, the triple therapy group experienced fewer cases of hypoglycemia and less weight gain.

While the study’s endpoints were established before the adoption of the current consensus definition of drug-free remission, its findings were instrumental in shifting treatment paradigms from sequential monotherapy toward early, intensive combination therapy. The results highlight the importance of establishing a favorable metabolic environment through multi-targeted intervention to achieve long-term glycemic stability—a principle now central to modern remission strategies. Future research should build on this foundation by evaluating head-to-head comparisons of triple oral therapy and novel agent combinations, with contemporary remission criteria as primary endpoints.

##### 3.5.2.2 Combination therapy (metformin+GLP-1+SGLT2i)

The combination of SGLT-2i, GLP-1RAs, and metformin shows excellent performance in weight reduction, reduction in hypoglycemic occurrences, and lowering of death rates from cardiovascular issues and all causes. However, no clinical studies have focused on diabetes remission as the primary outcome. Future research could assess its effects on diabetes remission in newly diagnosed or early-stage patients with T2DM.

### 3.6 New target drug research

#### 3.6.1 GPR40 agonists

G protein-coupled receptors (GPCRs) are important drug targets for T2DM (59). GPR40, a GPCR highly expressed in the pancreas (60), is activated by free fatty acids (FFAs). FAs binding to GPR40 trigger inositol trisphosphate (IP3) production and release intracellular calcium (+2) from the endoplasmic reticulum (ER). Additionally, GPR40 activation promotes calcium (+2) influx via calcium channels. Binding may increase intracellular cAMP levels, inhibiting ATP-sensitive potassium (KATP) channels and enhancing calcium influx. The elevated calcium (+2) can boost glucose-stimulated insulin secretion (GSIS), making GPR40 a potential target for T2DM remission (61–63).

#### 3.6.2 GPR119 agonists

GPR119 agonists, belonging to the GPCR family, are chiefly located in pancreatic β-cells and intestinal L-cells. They increase GLP-1 (64) and GIP levels, promoting insulin secretion and regulating glucose homeostasis. Both GPR40 and GPR119 agonists are crucial for diabetes treatment by regulating insulin secretion, reducing the risk of hypoglycemia, and providing new drug targets. Future research should focus on developing dual-target agonists (e.g., GPR40/GPR119 dual agonists) to explore new possibilities for T2DM remission therapies (**Table 1**).

### 3.7 Outlook on reversing diabetes with non-insulin antihyperglycemic drugs

Growing evidence shows early non-insulin antihyperglycemic drug intervention can achieve T2DM remission. However, several questions remain unanswered. Future research should focus on: (1) optimizing remission rates: related research has indicated that the natural remission rate of untreated T2DM is extremely low (65, 66). Low natural and achieved remission rates indicate the need to develop strategies beyond traditional glycemic control to reverse diabetes. (2) Extending remission duration: as remission rates decline over time (the Look AHEAD study; 67), further research should identify influencing factors, assess the synergistic effects of combining insulin with non-insulin drugs (such as GLP-1RAs and SGLT2i), and evaluate novel management models and digital health in delaying remission duration. (3) Developing models: there is an urgent need for models integrating genetic susceptibility, pancreatic function reserve (C-peptide dynamics), and metabolic memory effects, combined with machine learning for personalized remission probability assessment. (4) Confirming long-term benefits: decade-long cohort studies are required to determine the correlation between diabetes remission and microvascular (kidney/retina), macrovascular (ASCVD) complications, and all-cause mortality, focusing on the “metabolic memory” time window and intervention thresholds. (5) Expanding beyond T2DM subtypes: preliminary evidence suggests remission potential in special diabetes types. For example, vitamin D3 + sitagliptin may prolong remission among young patients with type 1 diabetes (68), and verapamil may improve C-peptide levels (69) among patients with type 1 diabetes. RCTs are required to corroborate these. Evidence-based strategies for remission in gestational diabetes and monogenic diabetes also need to be established.

### 3.8 Considerations for clinical translation

The clinical translation of emerging therapies for T2DM requires careful consideration of several key factors. Among these, the cost and accessibility of newer agents—such as GLP-1RAs and SGLT2is—pose significant barriers to their widespread adoption, particularly in low- and middle-income countries. The financial burden associated with these medications may limit patient access and hinder broader implementation efforts.

Patient adherence to injectable therapies, including GLP-1Ras, can be compromised by issues such as discomfort during administration, storage requirements, and the frequency with which these drugs must be administered. Adverse effects further complicate treatment protocols. GLP-1RAs are commonly associated with genitourinary infections, while SGLT2is can increase the risk of genitourinary infections. These side effects necessitate proactive management and underscore the importance of comprehensive patient education to ensure safe and effective use of these medications. There is a clear need for further health economic analyses and real-world studies to assess the long-term cost-effectiveness of these remission-inducing treatments. Such investigations should also consider their impact on patients’ quality of life and explore strategies to promote equitable access across diverse populations.

## 4 Non-glucose-lowering drugs for T2DM: progress, selection, and confusion

### 4.1 Metabolic Modulators: Multitarget Interventions and Potential for Diabetes Remission

In T2DM management, metabolic modulators should target weight loss, glycemic control, uric acid reduction, lipid regulation, anticoagulation, and homocysteine lowering. Correcting these metabolic disorders may promote diabetes remission by reducing insulin resistance and protecting β-cell function (**Table 2**), thereby creating a conducive metabolic environment for remission.

**Table 2:** Potential for diabetes remission using non-antihyperglycemic drugs and interventions.

Drug/intervention	Mechanism of action	Remission outcomes (key data)	References
Antihypertensive drugs (ARBs/ACEIs)	Improving insulin sensitivity. Telmisartan (ARB): Partially activates peroxisome proliferator-activated receptor gamma (PPAR-γ) (target of TZD antidiabetic agents) without TZD-associated edema.	In the valsartan group, the risk of new-onset diabetes was reduced by 23%; a meta-analysis showed that a 5 mm Hg reduction in systolic blood pressure could reduce the risk of diabetes by 11%.	(70)
Testosterone replacement therapy (TTh) in men	Improving insulin resistance and increasing muscle mass.	In the TTh group, HbA1c levels decreased, and 90% of patients achieved normal glycemic regulation (HbA1c < 5.7%).	(71)
Hormone replacement therapy (HRT) in women	Regulating glucose metabolism in postmenopausal women.	Meta-analyses: Reduces risk of new-onset T2DM	(72)
Jinlida granules	Regulate glucose and lipid metabolism, and improve insulin resistance.	43.8% of IGT patients restored normal blood glucose levels (vs. 6.9% in the control group).	(73)
Tianqi antidiabetic capsules	Delays IGT progression to T2DM.	Diabetes risk decreased by 32.1% (HR = 0.68):63.13% restored normal glucose tolerance.	(74)
Orlistat (weight-loss medication)	Reducing the hydrolysis of intestinal triglycerides and decreasing the absorption of free fatty acids may increase the secretion of GLP-1 and GIP, thereby improving insulin release.	12 months: Weight loss (-3.89% vs. -1.27%, *P* < 0.001), HbA1c reduction (-0.62% vs. 0.27%, *P* < 0.002) vs. placebo.- Lowers fasting glucose, insulin levels, and antidiabetic drug doses.- No documented diabetes remission rates (HbA1c < 6.5% without antihyperglycemic medications).	(75, 76)
Phentermine/topiramate	Suppress appetite and reduce energy intake	In patients with T2DM, HbA1c decreased by 0.4% (*P* < 0.05).	(77)
Naltrexone/bupropion	Bupropion stimulates POMC neurons, while naltrexone blocks the opioid receptor-mediated feedback inhibition, thereby enhancing the activity of POMC neurons and improving control over food cravings.	At 28 weeks, body weight decreased by 6.5% from baseline, and the rate of weight loss of ≥ 5% was significantly higher than that of the placebo (*P* < 0.001).	(78)
Vitamin D	The presence of proteins involved in gene regulation, activation of transcription factors, regulation of intracellular calcium concentration, and enhancement of insulin receptor sensitivity.	After supplementation, the risk of new-onset diabetes decreased. In those with baseline deficiency, HbA1c decreased.	(79, 80)
Vitamin K2 (menaquinone-7)	Key cofactor for γ-carboxylation of osteocalcin (OC); OC promotes insulin secretion and improves insulin sensitivity.	After 12 weeks of supplementation, HbA1c and FPG were significantly reduced; long-term effects need to be verified.	(81)
Quercetin (natural flavonoid)	Antioxidant and glucose-lowering effects.	In the quercetin group, insulin and blood glucose levels were significantly improved; the risk of metabolic syndrome was reduced in animal models.	(82)

ARB, angiotensin receptor blocker; ACEI, angiotensin converting enzyme inhibitors; IGT, Impaired glucose tolerance; HbA1c, glycated hemoglobin; T2DM, type 2 diabetes mellitus; GLP-1, glucagon-like peptide-1; GIP, glucose-dependent insulinotropic polypeptide; POMC, hypothalamic pro-opiomelanocortin; FPG, fasting plasma glucose

#### 4.1.1 Weight-loss medications: bridging weight management and diabetes remission

Achieving effective weight reduction is fundamental to improving insulin sensitivity and β-cell function, making it an essential approach for attaining T2DM remission. Several FDA-approved anti-obesity medications (83), originally developed for managing obesity, contribute significantly to this objective by promoting substantial and sustained weight loss, thus providing a clinically viable option for facilitating diabetes remission.

##### 4.1.1.1 Orlistat

Orlistat, approved in 1999 for the treatment of obesity, acts as a pancreatic lipase inhibitor, reducing the absorption of FFA by inhibiting triglyceride hydrolysis in the gut (75, 84). Orlistat induces 2.8–4.8% average weight loss but often causes gastrointestinal side effects (75, 76). After 12 months, the orlistat group exhibited significantly greater weight (−3.89% vs. −1.27%, *P* < 0.001) and HbA1c (−0.62% vs. 0.27%, *P* < 0.002) reductions than did the placebo group, as well as lower fasting glucose, insulin levels, and antidiabetic drug doses. In overweight/obese patients with T2DM with poor metabolic control, orlistat can optimize glycemic control, facilitate weight loss, and reduce cardiovascular risk factors. Nonetheless, existing studies have not documented remission rates for diabetes (e.g., HbA1c < 6.5% without antihyperglycemic medications).

Although current studies do not directly report diabetes remission rates, defined by an HbA1c < 6.5% without antihyperglycemic drugs, their demonstrated benefits in improving insulin resistance and β-cell function have established a critical metabolic foundation for achieving diabetes remission following more intensive interventions.

##### 4.1.1.2 Phentermine-topiramate

Approved by the FDA in 2012 for obesity treatment (75), phentermine is a noradrenergic agent that reduces appetite and food intake by enhancing the release of norepinephrine and blocking its reuptake. The exact mechanism of topiramate’s weight-loss effect is unclear, but it may involve appetite regulation and reduced energy intake (85). The SEQUEL study documented significant weight reduction, with a higher proportion of participants in the phentermine-topiramate group achieving ≥ 5%, ≥ 10%, ≥ 15%, and ≥ 20% weight loss. This treatment decreased FPG and insulin levels and improved insulin sensitivity. In non-T2DM individuals, it reduced the annualized incidence of T2DM. In patients with T2DM, it significantly lowered HbA1c by 0.4% and 0.2% at 108 weeks compared to placebo, without increasing antihyperglycemic medication needs, whereas the placebo group required additional drugs (77) (large-scale RCT [*n* = 676, 108-week follow-up]).These sustained metabolic improvements, coupled with a “drug reduction” effect, suggest a potential to induce and sustain diabetes remission.

##### 4.1.1.3 Naltrexone/bupropion

Obtained FDA approval in 2014 (75), the naltrexone/bupropion combination stimulates hypothalamic pro-opiomelanocortin (POMC) neurons via bupropion and blocks opioid receptor feedback with naltrexone, enhancing POMC neuron activity to control impulsive eating (86). The COR-II clinical trial (78) (large-scale RCT [*n* = 1 496, 56-week follow-up]) confirmed that the combination led to a 6.5% weight reduction from baseline, after 28 weeks (vs. 1.9% with placebo, *P* < 0.001), sustained at 56 weeks (−6.4% vs. −1.2%, *P* < 0.001). There was a significantly higher rate of ≥ 5% weight loss compared to the placebo group (at 28 weeks: 55.6% vs. 17.5%; at 56 weeks: 50.5% vs. 17.1%, both *P* < 0.001). This combination also improved cardiovascular metabolic risk markers, quality related to weight, and control of eating. The most common adverse event, nausea, was mild-to-moderate and transient. Given its significant weight loss, metabolic benefits, and favorable safety profile, naltrexone/bupropion may become a novel therapy for achieving remission in T2DM.

#### 4.1.2 Antihypertensive drugs: synergy and optimization in diabetes remission strategies

Selecting the optimal antihypertensive agents is critical for managing T2DM remission in patients with hypertension. Different drug classes can exert synergistic, neutral, or adverse effects on remission, depending on their impact on insulin sensitivity and β-cell function.

##### 4.1.2.1 ARBs and ACEIs: potential glycemic benefits

Angiotensin-converting enzyme inhibitors (ACEIs) and angiotensin II receptor blockers (ARBs) are widely recognized as complementary agents in strategies for T2DM remission, largely due to their potential to improve insulin sensitivity. This positioning is supported by a meta-analysis showing that both drug classes significantly reduce the risk of new-onset diabetes (87).

In the VALUE trial, individuals taking valsartan had a 23% reduced risk of new-onset diabetes compared to those on amlodipine (70). Within the ARB group, telmisartan is distinguished by its unique capacity to partially activate PPAR-γ—the primary target of TZD antidiabetic agents. This mechanism suggests that, in addition to its antihypertensive effects, telmisartan may directly help reduce insulin resistance and decrease the likelihood of new-onset diabetes, without causing the typical TZD-associated side effect of edema. These findings suggest that ARBs may offer metabolic protection beyond blood pressure control, helping create favorable conditions for remission in patients with T2DM.

##### 4.1.2.2 Calcium channel blockers (CCBs): a metabolically neutral choice

Calcium channel blockers (CCBs) are widely regarded as a metabolically neutral antihypertensive agent suitable for patients with hypertension and comorbid T2DM. Studies on calcium channel blockers (CCBs) and human pancreatic β-cells show that CCBs such as nifedipine and verapamil cannot shield β-cells from ER stress and saturated fatty acid-induced apoptosis. This contradicts rodent model findings, indicating CCBs have limited value in preventing and treating T2DM, especially when saturated fatty acids trigger β-cell apoptosis (88). Due to their minimal impact on glycemic control, CCBs offer a reliable therapeutic option for effective blood pressure management without interfering with diabetes remission efforts.

##### 4.1.2.3 Thiazide diuretics and beta-blockers: potential adversaries in remission strategies

Traditional thiazide diuretics, such as hydrochlorothiazide, and non-selective beta-blockers, like atenolol and metoprolol, not only lower blood pressure but can also negatively affect glucose and lipid metabolism, presenting obstacles to diabetes remission efforts. According to a systematic review, thiazide diuretics may dose-dependently exacerbate insulin resistance, elevate uric acid levels, and cause hypokalemia, thereby increasing the risk of new-onset T2DM by 20–30% (89. Non-selective beta-blockers further complicate remission strategies by inhibiting insulin secretion through β2-receptor blockade, increasing triglyceride levels, promoting weight gain, and masking the adrenergic warning symptoms of hypoglycemia. This masking effect increases the risk of glycemic rebound when discontinuing the drug.

In contrast, third-generation α1/β-blockers, such as carvedilol, have shown more favorable metabolic profiles. In the GEMINI trial (*n* = 1,235), carvedilol was associated with stable HbA1c levels and improved HOMA-IR, leading to recommendations for its use in patients with high metabolic risk. Consequently, when designing combination remission regimens for hypertensive patients—such as “oral agents + insulin” or “oral agents + GLP-1RA”—it is advisable to avoid traditional thiazide diuretics and non-selective beta-blockers. If these medications are necessary, clinicians should limit doses and closely monitor metabolic parameters to prevent compromising β-cell function recovery and the potential for drug-free remission.

Future clinical research should further investigate the relationship between blood pressure control and diabetes remission. Key areas of study include the effects of specific antihypertensive medications, the impact of varying degrees of blood pressure reduction on glycemic metabolism, and the mechanisms linking blood pressure management to diabetes remission. Developing best practices for integrating blood pressure control into diabetes remission strategies will support better metabolic health and optimize remission outcomes.

### 4.2 Sex hormone therapies: potential and considerations as adjunctive strategies for diabetes remission

Hormone replacement therapy (HRT) may serve as an adjunctive strategy in the comprehensive management of T2DM remission, particularly by ameliorating insulin resistance and preserving β-cell function. While most supporting evidence is derived from observational studies, HRT is considered more effective at creating a favorable metabolic environment rather than directly inducing remission. Clinical evidence shows that serum testosterone levels correlate positively with insulin sensitivity and negatively with insulin resistance. Testosterone replacement therapy can reduce HOMA-IR (90), decrease fat mass, and increase muscle mass, all of which have a positive impact on metabolism (91). Additionally, testosterone upregulates GLUT4 expression, promoting insulin-mediated glucose uptake (92) and protects pancreatic β-cells from inflammation, thereby maintaining or enhancing insulin secretion (90).

#### 4.2.1 Testosterone therapy in men

Men with T2DM and prediabetes often have low testosterone levels and hypogonadism. A meta-analysis (93) showed that testosterone therapy (TTh) in men with T2DM enhanced fasting glucose, HbA1c, fasting insulin, HOMA-IR, and fat quality. An 8-year prospective study (*n* = 316) (71) found that TTh reduced HbA1c, with 90% achieving normal glycemic regulation (HbA1c < 5.7%), and 40.2% of untreated individuals progressing to T2DM (HbA1c > 6.5%). A review of RCTs and real-world evidence (RWE) studies confirms that TTh significantly improves insulin resistance and glycemic control (94). Long-term TTh prevents progression to T2DM in men with low gonadotropin levels and prediabetes. However, these observational findings may have baseline imbalances between the TTh and untreated groups, necessitating validation through RCTs. Based on current evidence, TTh cannot be recommended as an independent or universal strategy for diabetes remission. Its use should be restricted to male patients with T2DM who also present with symptomatic hypogonadism, aiming primarily to improve metabolic parameters and potentially enhance the effectiveness of other core remission therapies, such as lifestyle interventions or GLP-1 receptor agonists.

#### 4.2.2 Hormone replacement therapy in women

Studies and meta-analyses have demonstrated that HRT significantly reduces insulin resistance and the risk of new-onset T2DM (72). A recent systematic review focusing on postmenopausal women with type 1 and type 2 diabetes found that hormone therapy (HT) significantly lowers HbA1c by 0.56% and fasting blood glucose by 1.15 mmol/L (95). HT was found to have a neutral to beneficial effect on glycemic control in this population. Despite these metabolic benefits, HRT carries risks, including a slight increase in the likelihood of stroke and breast cancer (95). Clinical decisions should carefully weigh these risks against benefits, taking into account patient age (early menopause, < 60 years or within 10 years, may confer more benefits), cardiovascular risk, and family history of breast cancer. Currently, HRT is not advised as a strategy for diabetes remission and should be individualized based on guidelines. While HRT can be considered for its primary indications, its metabolic benefits may serve as an adjunct in select cases.

### 4.3 Traditional Chinese medicine: modern challenges of traditional medicine

Traditional Chinese medicine and its active ingredients have demonstrated effectiveness in managing metabolic syndrome by lowering blood glucose, blood pressure, and fat accumulation (96, 97). These mechanisms lay the foundation for their potential role in influencing the course of T2DM, thereby reducing the risks of cardiovascular diseases, diabetes, and obesity. Although current evidence does not directly establish TCM’s ability to induce diabetes remission, the metabolic improvements achieved through its multi-target actions suggest promise for future exploration within T2DM remission strategies.

#### 4.3.1 Monomeric preparations

Natural monomeric compounds, with their well-defined chemical structures and diverse biological activities, provide a valuable resource for drug discovery and development. Over 5,000 flavonoid compounds exist in herbs. Quercetin, an important flavonoid widely found in berries, grapes, cherries, apples, red onions, peppers, citrus fruits, coriander, cauliflower, and tea, shows multiple metabolic regulatory effects, such as lowering blood glucose, reducing weight (98), lowering blood lipids (99), reducing blood pressure (100), and providing cardiovascular protection. Cross-sectional studies in Chinese populations have shown a negative correlation between quercetin intake and T2DM prevalence, suggesting its potential protective role (101). Future research should conduct rigorous clinical trials to evaluate the potential effects of quercetin on T2DM remission.

Despite existing medications for obesity and T2DM, concerns regarding adverse effects persist. This has led to increased interest in developing safer, effective alternative therapies from natural sources, particularly seaweed, which is rich in bioactive components. Studies suggest that seaweed-derived compounds such as fucoxanthin, fucoidan, and astaxanthin possess antioxidant, anti-inflammatory, antibacterial, neuroprotective, and cardioprotective properties (102). These compounds may improve metabolic syndrome by increasing adiponectin, decreasing leptin, reducing insulin resistance, elevating insulin levels, and blocking calcium channels (103–106). However, most evidence is derived from *in vitro* and animal studies, with limited clinical research. Future work should emphasize rigorous clinical trials for efficacy and safety validation, facilitating the development of seaweed-based drugs, functional foods, or dietary supplements, offering a new auxiliary therapeutic avenue to facilitate diabetes remission.

#### 4.3.2 Traditional Chinese medicine compound preparations

Traditional Chinese medicine formulas have demonstrated potential in delaying the progression from impaired glucose tolerance (IGT) to T2DM. In a single-center, open-label, phase II pilot RCT (*n* = 61, 12-week follow-up) Jinlida treatment significantly reduced 2-hour plasma glucose, HbA1c, and HOMA-IR. Normal blood glucose levels were restored in 43.8% of Jinlida-treated patients, compared to 6.9% in the control group, with diabetes incidence at 6.2% and 17.2%, respectively (73). Another multicenter, double-blind, phase IIb RCT (*n* = 389, 12-month follow-up) found that after 12 months, Tianqi Jiangtang capsules reduced diabetes progression to 18.18%, compared to 29.32% (*P* < 0.01), and increased normal glucose tolerance restoration to 63.13%, compared to 46.60% (*P* < 0.001). Cox regression analysis indicated a 32.1% lower risk of diabetes in the Tianqi group (74). These findings highlight the potential of Jinlida granules and Tianqi Jiangtang capsules in diabetes prevention. Future research should explore their long-term effects on blood glucose homeostasis, weight changes, β-cell function, and diabetes remission rates in patients with confirmed T2DM to determine their clinical value.

### 4.4 Vitamins and minerals

#### 4.4.1 Vitamin D

Vitamin D (1,25-dihydroxycholecalciferol) is a fat-soluble vitamin essential for calcium and phosphorus metabolism and bone health. It exerts autocrine, endocrine, and paracrine effects in various tissues (107). Regarding glucose metabolism, it mimics insulin action via mechanisms such as gene regulation, transcription factor activation, modulation of intracellular calcium concentration, and enhanced insulin receptor sensitization (108). Large-scale clinical studies, including the Tromsø study (79), the D2d study (109), and the DPVD study (110), indicate that vitamin D supplementation can reduce the probability ofT2DM and increase the likelihood of returning to normal glucose regulation, with no significant safety concerns (111). Meta-analyses have confirmed that vitamin D supplementation significantly improves FPG, HbA1c, HOMA-IR, and fasting insulin levels in patients with T2DM, particularly in those with low baseline vitamin D levels, overweight status, or HbA1c ≥ 8%, with better results following short-term, high-dose regimens. In summary, vitamin D can delay the progression of prediabetes and serve as an adjunct therapy for T2DM (80).

While metabolic outcomes are promising, the clinical applicability of vitamin D supplementation depends on baseline vitamin D status, dosing strategies, and interindividual response variability. Cost and adherence to long-term supplementation may also impact outcomes, particularly in resource-limited populations. Future trials should explore the feasibility of routine vitamin D screening and supplementation and investigate its synergistic effects with core remission therapies, such as intensive lifestyle intervention, to clarify its adjunctive value within multimodal remission frameworks.

#### 4.4.2 Vitamin K

Vitamin K is a key cofactor of the γ-carboxylation of osteocalcin (OC), a γ-carboxyglutamic acid (GLA) protein secreted by osteoblasts. OC promotes insulin secretion and improves insulin sensitivity, thereby regulating glucose metabolism (112, 113). Two prospective cohort studies show a negative correlation between dietary vitamin K and T2DM risk (114), a large-scale prospective cohort (*n* = 38,094, median 10.3-year follow-up) (115), and a prospective cohort embedded in the multicenter PREDIMED-Diabetes RCT (*n* = 1,069, median 5.5-year follow-up). A single-center, double-blind, phase II RCT (*n* = 68, 12-week follow-up) found that 12 weeks of daily 180 μg vitamin K2 (MK-7) supplementation significantly reduced FPG and HbA1c, and improved blood glucose target achievement rates. Although insulin levels and HOMA-IR improved within the vitamin K2 group, between-group differences were not statistically significant, and vitamin K2 had no significant impact on lipid levels (81). Recent findings show that vitamin K2 may enhance blood sugar control via an OC-dependent pathway, but limitations include a short intervention period (12 weeks) and a lack of direct correlation analysis between OC carboxylation and metabolic improvement. Future research should extend the follow-up and include OC carboxylation rate and dynamic pancreatic function assessments to determine the role of vitamin K in diabetes remission and clinical value (**Table 2**).

## 5 Long-term durability of remission

The principal benefit of T2DM remission is its long-term sustainability. However, current research reveals notable differences between short-term (< 1 year) and long-term (≥ 1 year) remission outcomes, necessitating a thorough analysis that considers factors such as follow-up duration, patients’ baseline characteristics (e.g., disease duration, BMI), and the specific intervention regimens used (**Table 3**).

**Table 3:** Comparison of short- and long-term remission rates across pharmacological classes.

Pharmacological category	Short-term remission (Key data, < 1 year)	Long-term remission (Key data, ≥ 1 year)	References
Short-Term Intensive Insulin Therapy (SIIT)	1. Multicenter RCT (*n* = 412, The BMJ 2024): Newly diagnosed T2DM with severe hyperglycemia (mean HbA1c 11.0%) received 2–3 weeks of SIIT; 48% maintained HbA1c < 6.5% at 48 weeks (drug-free remission). 2. Medium-scale RCT (*n* = 124, 3-month follow-up): Patients with baseline BMI ≥ 25 kg/m² had higher remission rates and greater improvements in GIR/AIRins vs. lean patients.	Medium-scale RCT (*n* = 124, 2-year follow-up): No significant difference in remission rates between BMI ≥ 25 kg/m² group (43.1%) and lean group (39.8%, *P* > 0.05).	(17, 19)
Metformin	1. pivotal RCT: 12-week intensive intervention (lifestyle + basal insulin/GLP-1RAs + metformin) achieved remission rates at 24–36 weeks approximately double those of the standard care group (primary outcome: HbA1c < 6.5% off all antihyperglycemic drugs). 2. No standalone short-term remission data; efficacy mainly reflected in combination with other therapies.	1. Small-scale RCT (*n* = 48, 4-year follow-up): After initial remission via short-term intensive insulin therapy, metformin (1000 mg/day) reduced hyperglycemia recurrence risk by ~70% vs. placebo, and extended median remission duration from ~10 months to 16 months (mechanism: improved β-cell function). 2. No standalone long-term remission data; serves as core for sustaining remission in combination strategies.	(27, 30, 32, 33)
SGLT-2 Inhibitors	1. REMIT-DAPA trial (medium-scale RCT, *n* = 154): Short-term intensive intervention (insulin + metformin + dapagliflozin) showed relative risks for remission of 2.4, 2.1 at 36, 48 weeks vs. control; reduced relapse risk by 43%.	1. Combination therapy (12-week intensive intervention: lifestyle + basal insulin + lixisenatide + metformin): At 36 weeks after drug withdrawal, remission rate approximately doubled vs. standard care group (relative risk = 1.83); reduced diabetes relapse risk by 43%. 2. Sequential therapy (*n* = 129, 2-year follow-up): 53.0% cumulative remission rate vs. 31.8% in insulin-only group, but therapeutic advantage disappeared after exenatide discontinuation. 3. “Basal insulin + short-acting GLP-1RA” (exenatide, *n* = 109, 20-week follow-up): No significant improvement in β-cell function or long-term remission potential vs. basal insulin alone.	(27)
GLP-1 Receptor Agonists	1. Combination therapy (12-week intensive intervention: lifestyle + basal insulin + lixisenatide + metformin): At 24 weeks after drug withdrawal, remission rate approximately doubled vs. standard care group (relative risk = 1.92). 2. Sequential therapy (3-week intensive insulin pump + 12-week exenatide, *n* = 129): 68.2% cumulative remission rate at 1 year (definition: HbA1c < 7.0% off glucose-lowering drugs) vs. 36.5% in insulin-only group. 3. Monotherapy: No consensus-level evidence (no RCTs with primary endpoint of “HbA1c < 6.5% without pharmacotherapy for ≥ 3 months” as of June 2025).	Higher remission rate vs lifestyle or SU groups (*n* = 278, 18 months).	(44, 45)
TZDs (Pioglitazone)	57.9% achieved HbA1c < 6.5% (*n* = 325); maintained HbA1c < 6.2% longer post-discontinuation (*n* = 278).	No long-term remission data yet; weight loss up to 9.5kg (40 weeks).	(21)
Dual/Triple Agonists (Tirzepatide)	86% achieved HbA1c < 6.5% with 15mg dose (*n* = 478, 40 weeks); 95.3% normoglycemia in prediabetics (*n* = 2539).		(25, 26)
Triple Oral Therapy	HbA1c dropped from 11.43% to 6.17% at 3 months (*n* = 44).	36.3% maintained normoglycemia after SU withdrawal (*n* = 373, avg 4 months remission).	(51)

### 5.1 Short-term intensive insulin therapy

SIIT can deliver marked benefits in the initial months following administration, particularly for patients newly diagnosed with T2DM. A recent multicenter study comprised 412 patients with newly diagnosed T2DM and severe hyperglycemia (mean HbA1c: 11.0%), all of whom received SIIT for two to three weeks. In the control group, which only adopted lifestyle interventions without further medication, nearly half (48%) maintained their HbA1c below 6.5% after 48 weeks. Early SIIT was found to significantly improve β-cell function and insulin sensitivity, laying the foundation for long-term glycemic control without medication for some patients.

Further evidence from Wang et al. (19) showed that among 124 patients receiving SIIT therapy, those with a baseline BMI ≥ 25 kg/m² had a greater one-year remission rate (61.3%) versus lean patients (45.2%). However, this difference dissipated at 2 years (43.1% vs. 39.8%, *P* > 0.05), indicating that body weight influences short-term remission, whereas long-term outcomes may depend more on recovery of β-cell function. Most follow-up studies examining SIIT focus on patients with a disease duration of less than 2 years, with limited data available for those with a longer history (> 5 years). There is also insufficient information regarding the effect of intervention duration (e.g., 2 weeks vs. 3 months) on long-term remission.

### 5.2 Non-insulin antihyperglycemic therapies

Non-insulin glucose-lowering agents offer alternative long-term remission options for T2DM. Among these, GLP-1RAs have demonstrated the most promising long-term outcomes, while further data are needed for SGLT-2is.

#### 5.2.1 GLP-1RAs and combination therapy

Evidence from a two-year follow-up study by Shi et al. (45) involving 120 patients demonstrated that combination therapy with SIIT plus exenatide achieved a two-year remission rate of 53.0%, compared to 31.8% with SIIT alone (*P* < 0.001). Sustained weight loss (average reduction: 4.2 ± 1.5 kg) and improved β-cell function (HOMA-β maintained at 85.3 ± 12.6) were linked to long-term remission.

#### 5.2.2 SGLT-2 inhibitors

The Remission Evaluation of Metabolic Interventions in Type 2 Diabetes with Dapagliflozin study reported that short-term intensive metabolic intervention increases remission rates. The relative risks for diabetes remission at 36, 48, and 64 weeks were 2.4, 2.1, and 1.8, respectively; having reduced the relapse risk by 43% (27) (medium-scale RCT [n = 154, maximum follow-up 1.2 years]).

### 5.3 Non-glucose-lowering drugs

The adjunctive role of non-glucose-lowering agents in sustaining long-term remission is limited, with current evidence lacking consistency and robustness. Agents such as vitamins and sex hormones are primarily considered adjunctive or supportive interventions, and available data do not substantiate their efficacy for long-term remission.

#### 5.3.1 Testosterone replacement therapy

An 8-year observational cohort study by Yassin et al. (71) reported that 90% of 316 male patients treated with TTh maintained HbA1c levels below 5.7%. However, the study did not control for confounding factors, such as lifestyle interventions (e.g., weight control). Moreover, concerns regarding potential prostate-related risks limit the long-term application of testosterone, precluding its broader recommendation as a standard remission strategy.

#### 5.3.2 Vitamin D/K

A 12-week study of vitamin K2 revealed modest short-term improvements in glycemic control and did not provide data supporting remission beyond one year (81). Although a meta-analysis suggested that vitamin D may reduce the risk of developing T2DM, it did not specifically address long-term remission in those already diagnosed. Overall, findings suggest that non-glucose-lowering agents function only as adjuncts in the pursuit of long-term remission and cannot independently maintain sustained glycemic control (80).

## 6 Subgroup-specific benefits and regimen selection based on patient characteristics

Patients with T2DM exhibit varying responses to pharmacological remission regimens, largely influenced by factors such as disease duration, body weight, islet functional reserve, and comorbidities. Recognizing and understanding these subgroup-specific benefit profiles is critical to implementing effective, individualized remission therapy.

### 6.1 Stratification by disease duration

Patients newly diagnosed with T2DM, or those with a disease duration of less than two years, typically retain β-cell function (with HOMA-β values > 50). These individuals respond well to STII. Notably, in newly diagnosed patients with overweight/obesity (BMI ≥ 25 kg/m²), combination therapy with GLP-1RAs (e.g., semaglutide) and metformin has shown increased efficacy. A trial by Rosenstock et al. (25) demonstrated a one-year remission rate of 68.2% in this subgroup, which was significantly higher than the 45.5% rate observed in normal-weight patients. Furthermore, patients who achieved at least 5% weight loss experienced double the duration of remission.

Additionally, among patients with comorbid insulin resistance (HOMA-IR > 5), daily supplementation with 1000 IU of vitamin D was associated with an extension of remission by three to six months (80); however, these individuals did not achieve drug-free remission.

### 6.2 Stratification by body weight

#### 6.2.1 Patients with overweight/obesity (BMI ≥ 25 kg/m²)

Patients with overweight/obesity commonly experience pronounced insulin resistance. For this group, GLP-1RAs provide glucose-lowering and weight-reducing benefits. The SURPASS-1 trial demonstrated that participants with a BMI of 30 kg/m² or higher who received tirzepatide (15 mg) achieved a one-year remission rate of 86%, with an average weight loss of 9.5 kg, compared to a 78% remission rate and 7.0 kg weight loss in those with a BMI between 25 and 29.9 kg/m² (*P* < 0.05). SGLT-2is, such as dapagliflozin, are also suitable for patients in this subgroup, particularly those with mild-to-moderate renal impairment (estimated glomerular filtration rate [eGFR] 60–90 mL/min/1.73 m²).

#### 6.2.2 Patients with normal weight (BMI < 25 kg/m²)

Individuals with normal weight generally maintain normal insulin sensitivity, making the restoration of β-cell function a more critical factor for remission. Wang et al. (19) reported that newly diagnosed, normal-weight patients who received SIIT achieved a one-year remission rate of 51.2%, which was significantly higher than the 32.8% rate achieved with GLP-1RA monotherapy, without causing excessive weight loss. For those unable to tolerate injectable therapies, an oral regimen comprising metformin and SGLT-2i may be considered, with a one-year remission rate of 34.5%; this approach is particularly appropriate for older adults.

### 6.3 Stratification by comorbidities

#### 6.3.1 Comorbid cardiovascular or chronic kidney disease

In patients with coexisting cardiovascular disease (such as heart failure) or chronic kidney disease (CKD stages G1–G3), both remission efficacy and organ protection must be considered. The REMIT-DAPA trial found that individuals with T2DM and comorbid heart failure who received a regimen of insulin, dapagliflozin, and metformin had a 2-year remission rate of 38.7%, compared to 21.5% in those treated with insulin and metformin. Moreover, treatment with dapagliflozin reduced the risk of heart failure readmission by 42% (27). For patients with advanced CKD (G4–G5), SGLT-2is are contraindicated. In these cases, a combination of liraglutide and basal insulin is recommended, yielding a remission rate of 28.9% and a decreased risk of renal function decline (34).

#### 6.3.2 Males with low testosterone (<300 ng/dL) or postmenopausal females

Males with comorbid low testosterone levels (< 300 ng/dL) and postmenopausal females may benefit from adjunctive sex hormone therapy to enhance remission outcomes. In a study of 316 male participants, Yassin et al. (71) found that the one-year remission rate was higher in those who received SIIT plus TTh at 58.3%, compared to 39.1% with SIIT alone; however, this study excluded individuals with a history of prostate cancer.

## 7 Conclusion and future perspectives

Achieving remission of T2DM has become a crucial objective in diabetes treatment. This review summarizes three main drug intervention strategies: (1) SIIT rapidly alleviates glucolipotoxicity, reshapes β-cell function, and induces sustained glycemic remission in some patients; (2) Novel non-insulin medications (such as SGLT-2i, GLP-1RAs, and multi-receptor agonists) show remission potential via multitarget regulation, including metabolic restructuring, β-cell protection, and weight loss; (3) Non-antihyperglycemic drugs (such as vitamin D/K, sex hormones, traditional Chinese medicine) provide adjunctive therapeutic options for diabetes remission by modulating metabolism and inflammatory microenvironments. Additionally, combination therapy strategies (e.g., insulin combined with OHA, multitarget oral drug combinations) are crucial for improving remission rates and durations. However, current research has limitations: long-term remission rates, treatment durability, and the optimization of individualized strategies require further study. Additionally, the mechanisms of different drugs in T2DM remission, particularly the potential mechanisms and long-term safety of non-antihyperglycemic drugs, require further clinical validation.

To guide rational clinical application of pharmacological strategies for T2DM remission, supporting studies can be further classified according to the strength of their evidence and evaluated based on their relevance to clinical practice.

High-quality large-scale RCTs: Findings from robust RCTs, such as the tirzepatide SURPASS series and the GLP-1RA LIBRA trial, offer direct guidance for clinical decision-making. Regimens including 15 mg tirzepatide monotherapy and liraglutide are particularly recommended for newly diagnosed patients who retain adequate β-cell function (HOMA-β ≥ 40%). These strategies have consistently demonstrated high rate of remission, with 81–86% of patients achieving HbA1c ≤ 6.5%, alongside acceptable safety profiles.

Moderate-quality cohort studies: Results from long-term cohort studies can inform clinical practice but must be applied with caution. For instance, TTh may be beneficial for men with T2DM and hypogonadism (serum testosterone < 8 nmol/L), but should be avoided in patients with prostate cancer or severe cardiovascular disease. This underscores the importance of tailoring treatment decisions to individual patient characteristics, such as gonadal function and comorbidities.

Low-quality small-sample studies: Preliminary data from small-scale studies should be regarded as avenues for future investigation rather than immediate clinical application. These interventions currently lack large-scale validation supporting the durability of remission (e.g., sustained HbA1c control for ≥ 2 years) and long-term safety (e.g., long-term herbal medicine toxicity), and thus are not recommended for routine clinical remission protocols.

Future research should focus on: (1) The “time window” for diabetes remission: Early intervention (e.g., within the first year of diagnosis) may reverse β-cell dedifferentiation and insulin resistance to achieve higher remission rates, whereas late-stage patients may need metabolic surgery or potent multitarget drugs. Research should identify the reversible critical point of metabolic memory to define optimal intervention timing. (2) Optimizing combination therapy: traditional stepwise treatment may miss remission opportunities, whereas early triple therapy (e.g., metformin + GLP-1RA + SGLT2i) or targeted intensification strategies (e.g., SIIT followed by GLP-1RA) may maximize synergistic effects. Head-to-head trials should compare long-term benefits of different combination regimens. (3) Systemic metabolic regulation beyond glycemic control: diabetes remission requires attention regarding comorbidities such as weight, fatty liver, and cardiovascular risk. Non-antihyperglycemic approaches such as vitamin D/K and anti-inflammatory drugs may enhance remission durability by improving the metabolic microenvironment.

Future research directives should focus on (1) developing subgroup-specific predictive models—such as designing a scoring system for remission probability that incorporates factors like disease duration, BMI, and HOMA-β—that can offer clinicians more accurate guidance for selecting treatment plans. (2) Designing specialized trials targeting special subgroups (e.g., older adults with a long disease duration, patients with comorbid complex diseases) to fill the current evidence gaps. (3) Investigating the heterogeneous mechanisms of post-treatment effects and exploring strategies to maintain long-term remission. (4) Developing new drugs, such as long-acting formulations (e.g., GLP-1RA with half-yearly dosing); smart drug delivery systems (e.g., glucose-dependent release); novel combinations; non-peptide oral agonists (e.g., dual-target drugs for GPR40/119); and gene therapies targeting β-cell regeneration. These innovations may improve remission rates and reshape T2DM treatment. (5) Establishing standardized diabetes remission clinics that integrate pharmacotherapy, digital therapies (e.g., AI-based dietary management), and metabolic monitoring. Longitudinal cohort studies should be conducted to verify the association between remission and microvascular/cardiovascular outcomes and determine the duration threshold of “metabolic memory.”

Future efforts should focus on translating these strategies into real-world applications to transition T2DM remission from theory to clinical practice, ultimately reducing medication burden and improving long-term prognosis. However, it is important to acknowledge that current evidence focuses primarily on efficacy under experimental conditions, leaving notable gaps in knowledge regarding the durability of remission, predictive factors for sustained success, and the mechanisms underlying long-term maintenance. The effective translation of T2DM remission strategies into clinical practice requires not only the development of more advanced pharmacologic and therapeutic strategies but also a comprehensive assessment of cost-effectiveness, accessibility, patient adherence, and safety. Accordingly, future research should extend beyond mere exploration of efficacy and actively advance health economic evaluations, implementation research, and equity-oriented strategy practice. Integrating RWE, cost-effectiveness analyses, and considerations of accessibility into core evaluation frameworks is crucial to positioning diabetes remission as an attainable, sustainable, and scalable standard of care.
